# Exercise-Trained Men and Women: Role of Exercise and Diet on Appetite and Energy Intake

**DOI:** 10.3390/nu6114935

**Published:** 2014-11-10

**Authors:** Stephanie M. Howe, Taryn M. Hand, Melinda M. Manore

**Affiliations:** School of Biological and Population Health Sciences, Nutrition and Exercise and Sport Science, Oregon State University, Corvallis, OR 97331, USA; E-Mails: howest@onid.oregonstate.edu (S.M.H.); taryn.hand@oregonstate.edu (T.M.H.)

**Keywords:** hunger, female athletes, diet, energy density, energy intake, amenorrhea

## Abstract

The regulation of appetite and energy intake is influenced by numerous hormonal and neural signals, including feedback from changes in diet and exercise. Exercise can suppress subjective appetite ratings, subsequent energy intake, and alter appetite-regulating hormones, including ghrelin, peptide YY, and glucagon-like peptide 1(GLP-1) for a period of time post-exercise. Discrepancies in the degree of appetite suppression with exercise may be dependent on subject characteristics (e.g., body fatness, fitness level, age or sex) and exercise duration, intensity, type and mode. Following an acute bout of exercise, exercise-trained males experience appetite suppression, while data in exercise-trained women are limited and equivocal. Diet can also impact appetite, with low-energy dense diets eliciting a greater sense of fullness at a lower energy intake. To date, little research has examined the combined interaction of exercise and diet on appetite and energy intake. This review focuses on exercise-trained men and women and examines the impact of exercise on hormonal regulation of appetite, post-exercise energy intake, and subjective and objective measurements of appetite. The impact that low-energy dense diets have on appetite and energy intake are also addressed. Finally, the combined effects of high-intensity exercise and low-energy dense diets are examined. This research is in exercise-trained women who are often concerned with weight and body image issues and consume low-energy dense foods to keep energy intakes low. Unfortunately, these low-energy intakes can have negative health consequences when combined with high-levels of exercise. More research is needed examining the combined effect of diet and exercise on appetite regulation in fit, exercise-trained individuals.

## 1. Introduction

The regulation of appetite and energy intake are influenced by numerous hormonal and neural signals [1], including but not limited to diet and exercise [2,3], gastric motility [4], body size, temperature, and level of dehydration [5]. The integration of these signals within the brain, especially the hypothalamus, reflects the current energy status of the body, which is subsequently used to either stimulate or suppress appetite. Diet and exercise are two lifestyle behaviors that can influence appetite and energy intake; thus, ultimately altering energy balance. The impact of exercise on appetite and energy intake in athletes or exercise-trained individuals may be different from their sedentary counterparts. These individuals exercise at high-intensities on a regular basis and typically have normal body size and composition. In addition, the diets of exercise-trained individuals may further alter appetite and energy intake.

### 1.1. Impact of Diet

The type and quantity of food intake influences appetite and energy intake. Appetite-regulating hormones released from the gut in response to food intake provide feedback to the hypothalamus and, subsequently influence appetite. Thus, appetite is a tightly regulated process where many redundancies exist to regulate energy status. Despite the tightly controlled internal regulation of appetite, hedonistic factors, such as the sight and smell of food or circadian patterns (e.g., time of day), can also influence the desire to eat. As a result, food intake is the outcome of the integration of both internal and external factors. As evidenced by the weight management problem in over half of the population [6], internal factors signaling the energy status of the body are easily overridden by external factors influencing food intake. Thus, appetite and food intake are not simple behaviors, but rather a consequence of a myriad of internal and external signals.

Nutrient composition of the diet (e.g., macronutrient, alcohol and fiber content) also influences hunger and fullness and, thus, can alter energy intake. Research shows that consumption of a low-energy dense (ED) diet can increase satiety [7] and contribute to a reduction in total energy intake, which can ultimately result in weight loss [8]. For example, Rolls *et al.* [7] showed that a 25% reduction in dietary energy density resulted in a 24% reduction in energy intake in non-athletic women (19–45 years). Despite the reduction in energy intake, there were no differences in ratings of hunger and fullness compared to the control diet. This increased sense of fullness from the consumption of low-ED foods is attributed to their high-volume, fiber, and water content, and their low energy content per gram (kcal/g) [9,10]. Many female athletes and exercise-trained women are health conscious and/or concerned about their weight, thus, they self-selected foods lower in energy density (e.g., whole grains, fruits and vegetables, and low fat protein sources). While this is the dietary approach recommended by the 2010 Dietary Guidelines for Americans [11], for fit, highly active women with high-energy needs, a low-ED diet may not provide adequate energy to cover activities of daily living, reproduction, and exercise energy expenditure [12].

### 1.2. Impact of Exercise and Fitness Level

Another potential confounder of appetite is exercise, which can alter gut appetite-regulating hormones such as ghrelin, peptide YY (PYY), and glucagon-like peptide 1 (GLP-1) [13,14,15,16,17,18,19,20,21,22,23,24,25,26,27,28,29,30,31,32,33,34,35,36]. Although some controversy exists, a growing body of research indicates that an acute bout of exercise can transiently suppress appetite for 2–10 h post-exercise [17,20,37,38]. Further, the suppressive effects of exercise on appetite may be exercise intensity dependent, with greater suppression occurring after higher intensity exercise [16,22,27,32,37]. However, the research literature is still equivocal as to how appetite is influenced by the type, duration, intensity, and mode of exercise. In addition, subject characteristics (e.g., body fatness, fitness level, age or sex) differ and may further contribute to discrepancies in the research literature. Exercise-trained individuals exercise regularly and can exercise at higher intensities for longer periods of time compared to their sedentary counterparts. These two factors may result in post-exercise appetite suppression occurring frequently throughout the day, since athletes typically exercise daily and some exercise twice a day. Thus, more research is needed to understand the impact of various modes and intensities of exercise and fitness level on appetite in different populations.

### 1.3. Impact of Sex

Finally, sex also influences appetite. Although research suggests that fit individuals engaging in higher intensity exercise are better at compensating for energy expended during exercise, few studies focus on exercise-trained women [39]. In addition, the type and amount of food consumed may vary based on one’s perception of body size. For athletes and exercise-trained women preoccupied with their body weight and shape [40], eating a low-ED diet may help them regulate hunger, while still maintaining a low body weight. Thus, combining a low-ED diet with high levels of exercise, that may elicit post-exercise appetite suppression, only further increasing the risk of sub-optimal energy intakes in lean women. Ultimately, if energy intake does not match energy expenditure, these women are at increased risk for a number of health issues, including relative energy deficiency in sport (RED-S) [12], which encompasses the female athlete triad (amenorrhea, osteoporosis, eating disorders), stress fractures and suppressed immune response [41,42]. Thus, understanding the impact that both exercise and diet have on appetite, total energy intake, and energy balance is important for making health, weight management, and pre/post-exercise dietary recommendations to exercise-trained men and women.

This review focuses on exercise-trained men and women using only those studies clearly describing the fitness level of the participants (e.g., athlete, exercise-trained) and/or providing VO_2_max values indicating a high level of fitness. Because of these stringent criteria, some studies [22,23,43] that reported “active” participants were not included because their level of fitness and exercise training could not be confirmed based on the data provided. First, we examine the impact of exercise on hormonal regulation of appetite, subjective and objective measurements of appetite, and post-exercise energy intake. We then discuss the impact of exercise intensity, sex, and exercise training (e.g., beginning a fitness program) on appetite. Although extensive research has investigated the role of exercise on appetite and/or energy intake post-exercise in exercise-trained men [13,14,15,16,17,18,19,20,21,23,24,26,27,28,29,30,31,32,33,34,35,36,38,39,44,45,46,47], there is a lack of consensus and limited data for exercise-trained women [22,48]. The impact of exercise on subsequent energy intake and whether exercise-trained individuals totally compensate for energy expended during exercise is also reviewed. Next the impact that low-ED diets have on appetite and energy intake is discussed. Finally, the limited research on the combined effects of high-intensity exercise and low-ED diets is examined. This research has been done in exercise-trained women who are often concerned with weight and body image and may select low-ED foods to keep energy intakes low. Unfortunately, the resulting low-energy intakes can have negative health consequences when combined with high levels of exercise. More research is needed examining the combined effect of diet and exercise on appetite regulation in exercise-trained individuals.

## 2. Hormonal Regulation of Appetite

Appetite is a complex process involving numerous internal and external signals. Energy status within the body influences circulating hormones and feedback signals that regulate appetite and subsequent eating behavior [1]. Two types of hormones are involved with long- and short-term appetite regulation. Tonic circulating hormones, such as insulin and leptin, indicate long-term energy status and are involved with appetite suppression. Conversely, episodic hormones are released in response to feeding, or the anticipation of eating, and are involved in short-term appetite response. Most episodic or gut hormones, including cholecystokinin (CCK), GLP-1, and PYY, are involved in appetite suppression [1,38,49,50,51]. Only one episodic hormone, ghrelin, is involved with appetite stimulation [13,33,38,52,53]. Sex hormones, including estrogen and progesterone, can also influence appetite and food intake. Although other hormones may influence appetite, this review focuses on the hormones that are influenced by exercise. These hormones and their influence on energy intake are discussed below.

### 2.1. Insulin

Insulin is well known for its important role in nutrient uptake in the periphery following a meal. Insulin release from the β-cells of the pancreas stimulates glucose uptake by insulin dependent tissues, including the brain, adipose tissue, and skeletal muscle. In the brain, insulin receptors are widely distributed in the hypothalamus, indicating insulin may exert a central role. Although not totally understood, insulin signaling appears to be involved with appetite suppression [1].

### 2.2. Leptin

Leptin, a hormone produced by adipocytes, is released into the blood in a pulsatile fashion and transported to the hypothalamus, where it has an appetite-suppressing effect [54]. Circulating levels of total plasma leptin are proportional to total body adipose tissue and relay this information (e.g., level of stored energy) to the brain. Within the hypothalamus, leptin exerts anorexigenic effects by inhibiting orexigenic NPY/AgRP (Neuropeptide Y (NPY), Agouti-related peptide (AgRP)) co-expressing neurons and by stimulating anorexigenic POMC/CART (Pro-opiomelanocortin (POMC), cocaine- and amphetamine-regulated transcript (CART)) expressing neurons [1]. The net effect of the central actions of leptin is potent appetite suppression. A genetic leptin deficiency results in hyperphagia in obese individuals, which can be reversed with leptin administration. However, leptin administration to obese individuals without the genetic defect does not result in the same effects on appetite [1]. More recently, leptin has been found to play a role in short-term regulation of food intake. In addition to adipose tissue, a small amount of leptin is also produced by the stomach and involved with satiety along with other peptides released from the gut in response to food intake [55]. Thus, leptin appears to be involved in both long-term regulation of energy balance and short-term regulation of appetite suppression by modulating food intake [1].

### 2.3. Ghrelin

Ghrelin, mainly produced by the P/D1 cells of the stomach, is unique in that it stimulates appetite, rather than indicating satiety (see Table 1). In humans, the pre-prandial increase in ghrelin correlates with hunger scores, indicating that ghrelin acts as a meal-initiation signal in the short-term regulation of appetite [55]. Accordingly, weight loss, fasting, and hypoglycemia increase ghrelin mRNA expression and secretion [1]. Evidence also suggests that ghrelin has an inverse relationship with the energy content of food consumed, and subsequent rise in ghrelin prior to the next meal. However, the consumption of a low-ED diet for weight loss is associated with lower circulating concentrations of ghrelin, which may aid in weight loss or maintenance [56]. Ghrelin exists in two forms, acylated and deacylated, which determine its role in the body. Acylated ghrelin, the active form, is involved in the regulation of appetite [57]. In this review, mentions of ghrelin refer to the acylated, active form.

**Table 1:** Definitions of commonly used terms associated with eating behavior ^a^.

Term	Definition	Assessment
*Appetite*		
Hunger	Sensations that promote food consumption, including metabolic, sensory, and cognitive factors.	Questionnaires: subjective measure using a 100 mm Visual Analog Scale (VAS). Biomarkers: measurement of blood appetite-regulating hormones. Energy Intake: food choices, including the total amount of energy (kcals) and macronutrient composition including fat, carbohydrate, protein, and fiber.
Satiation	Sensations that determine meal size and duration.	
Satiety	Sensations that inhibit further eating and determine the inter-meal interval (e.g., the period of fasting between meals).	
*Energy Intake (EI)*		
Energy Density	Amount of energy (kcals) per gram (g) weight of food and expressed as kcal/g.	Food buffet presented to participants and the amount of food consumed and type of food selected are measured.
Macronutrient Composition	Amount of carbohydrates, fat, protein, alcohol and fiber in the foods consumed.	
Absolute EI	Total amount of energy consumed when offered a food buffet.	Food buffet presented and total kcals consumed recorded.
Relative EI	Total energy consumed post-exercise minus energy expended in exercise.	Food buffet presented, total kcals consumed recorded, and energy expended in exercise subtracted. This method accounts for the differences in exercise protocols.

^a^ Definitions adapted from Mattes *et al.* [58] and Rolls *et al.* [7].

### 2.4. Cholecystokinin

CCK is a gut peptide released from the small intestine in response to fat- or protein-rich chyme entering the duodenum. Circulating CCK acts centrally as a neurotransmitter signaling satiety. Peripheral administration of CCK can also inhibit food intake by reducing meal size and duration [1].

### 2.5. Glucagon-Like Peptide 1

GLP-1 is a peptide released from the L-cells of the small intestine in response to food intake. Locally, GLP-1 is involved in satiety by slowing gastric emptying, while circulating GLP-1 is a central appetite suppressor. GLP-1 peaks twice in response to food intake. The first peak is within 15 min after meal initiation in response to a neurohormonal reflex. The second larger peak occurs 90–120 min post-prandial and is a result of L-cell stimulation as the nutrients contained in food arrive in the gastrointestinal tract [51]. Thus, nutrients within the gut have the ability to stimulate GLP-1 both indirectly and directly [50]. GLP-1 is also a potent stimulator of glucose-dependent insulin release from the pancreas, involved in long-term energy balance [49].

### 2.6. Peptide YY

PYY is an amino acid peptide secreted from the L-cells of the small intestine. Circulating levels of PYY are low during fasting and rapidly increase following a meal. Two forms of PYY exist: PYY_1–36_ in the gut causes local effects on motility, while PYY_3–36_ released into the circulation reduces food intake centrally [1,38]. Similar to the other gut peptides, circulating PYY_3–36_ rises in proportion to the level of energy consumed. PYY_3–36_ increases within 15 min of food ingestion and remains elevated for 90 min following a meal. Thus, PYY is likely to be under neural control and released in response to nutrients present in the gut [59,60].

### 2.7. Sex Hormones

Sex hormones, including estrogen and progesterone, also influence appetite and food intake in women. During the late follicular phase of the menstrual cycle, estrogen is high while progesterone is low. It is during this phase that resting metabolic rate (RMR) is lowest as is energy intake. Conversely, during the late luteal phase of the cycle, progesterone is high while estrogen is low. RMR increases during this time and energy intake is at its highest [61,62]. Barr *et al.* [63] estimated that energy intake increases ~300 kcal/day during the luteal phase of the menstrual cycle. In female athletes with exercise associated menstrual dysfunction (e.g., amenorrhea) the body conserves energy by suppressing the reproductive cycle. This suppression is due to inadequate energy intake to cover both energy cost of exercise and reproduction [12,41,64].

Sex hormones can also interact with central and peripheral modulators of appetite to influence food intake. The precise mechanism is still unknown, but one hypothesis is that estrogen influences the satiation potency of CCK in the central hypothalamus during the peri-ovulation phase. Estrogen may also act on negative feedback controls related to food intake, specifically influencing meal size [65]. During menopause, in the absence of cyclic estrogen and progesterone, weight gain is common. Although other factors may play a role, the accumulation of body fat is attributed in part to estrogen deficiency. Thus, the onset of menopause is associated with increased body fat and decreased energy expenditure [66]. More research is needed to fully understand the interaction between reproductive hormones and appetite, and energy intake in women.

Although many appetite-regulating signals exist, recent studies have shown that ghrelin, GLP-1, and PYY are influenced by exercise [15,18,23,37,38]. Thus, this review focuses on the influence of exercise on these hormones.

## 3. Assessment of Appetite

The assessment of appetite includes both subjective and objective measurements. Objective assessment of appetite is done through the measurement of circulating appetite-regulating hormones (discussed above) [1]. However, changes in these hormones do not always translate into changes in energy intake. Thus subjective appetite assessment, or how hungry one feels, is typically measured using a Visual Analog Scale (VAS), which has been validated to record hunger, satisfaction, fullness, prospective food consumption, and desire to eat [67]. Further information about the VAS can be found elsewhere [67]. Taken together, subjective and objective measurements are used to better quantify appetite and understand the relationship to subsequent energy intake. See Table 1 for terms frequently used in the appetite regulation literature. Finally, it is important to distinguish between satiety and appetite (see Table 1). The increase in satiety seen with low-ED diets is due to the high volume, water and fiber content of food consumed, leading to a sense of fullness. Conversely, appetite includes the hormonal response and the hedonistic factors that influence energy intake. This distinction is important to keep in mind when examining how individuals regulate their energy intake.

## 4. Exercise, Appetite, and Gut Hormones

Although some controversy exists surrounding the impact of exercise on appetite, most studies using exercise-trained individuals have shown a suppressive effect of exercise on appetite. Highly fit individuals, who are accustomed to exercise, differ in their response to exercise compared to obese/overweight or sedentary individuals who do not exercise [39]. Below, we review the effect of acute exercise, both aerobic and resistance exercise, on appetite in exercise-trained individuals and whether one has a greater impact on appetite than the other. Research examining the impact of acute exercise on appetite in overweight/obese individuals is reviewed elsewhere [37,39,68]. We then examine the impact of exercise intensity and sex on appetite. Finally, the impact of initiating an exercise-training program (e.g., chronic exercise) on appetite in overweight/obese, sedentary individuals is reviewed. For the purposes of this review, we define exercise-trained males as having a VO_2_max ≥ 45 mL/kg/min and exercise-trained females having a VO_2_max ≥ 40 mL/kg/min (~60th percentile or above for 20–39 years; classified as in good aerobic fitness) [69] or described by the authors as “exercise-trained”, “athletes”, or “elite” individuals.

### 4.1. Acute Exercise (Aerobic and Resistance Exercise)

A growing body of research indicates that an acute bout of aerobic exercise suppresses appetite by decreasing levels of ghrelin, and increasing levels of PYY_3–36_ and GLP-1 [20,25,28,38]. Although the changes with aerobic exercise are transient, this suppression may be profound enough to decrease appetite and subsequent energy intake post-exercise. As shown in Table 2, when acylated ghrelin was measured post-exercise in exercise-trained men participating in exercise ranging from 30–240-min, all but one study showed a significant decrease. For those studies that measured PYY and GLP-1, all showed increases post-exercise in both men and women. Suppression of acylated ghrelin and elevation of PYY and GLP-1 indicate appetite blunting. Of the 24 studies described in Table 2, only four studies included women and only two studies focused only on female subjects. Thus, the influence of exercise on appetite in exercise-trained women is relatively unexplored. One reason for the limited research in women may be the impact of menstrual cycle on gastric emptying and gut hormones, thus, limiting when research can be done and requiring control of the menstrual cycle phase [70].

The influence of mode of endurance exercise (load (running) *vs.* non-load bearing (cycling)) on appetite has also been examined. A recent meta-analysis by Schubert and colleagues [39], found that exercise mode did not significantly impact the variation in study results, but could explain some of the differences. Exercise with greater metabolic and mechanical demand (weight-bearing exercise) showed greater appetite suppression. This meta-analysis did not limit types of participants and all were included, both men and women, as long as they were healthy and had no contraindications for exercise. Level of fitness was not used as a criterion for inclusion. Conversely, a recent study by Kawano and colleagues [18], which was not included in the review mentioned above, compared the effects of rope skipping (weight bearing) and cycling (non-weight bearing) on appetite in males (VO_2_max = 47.0 mL/kg/min). Results showed that rope skipping resulted in greater appetite suppression than cycling [18]. Thus, the impact of exercise mode on appetite suppression equivocal and more research is needed to answer this question.

Research has also examined the effect of an acute bout of resistance exercise on appetite in active and resistance trained individuals. Currently, only three studies have compared the impact of acute resistance *vs.* aerobic exercise on appetite-regulating hormones in exercise-trained individuals; however, it is not clear if participants were trained in both exercises modes [14,15,71]. In these studies, resistance and aerobic exercise ranged from 35–90 min [14,15,71]. Despite similar durations between conditions, aerobic exercise sessions resulted in greater energy expenditure compared to resistance exercise. Thus, changes in appetite and subsequent energy intake are difficult to compare. Nonetheless, all three studies reported little or no change in appetite-regulating hormones post-exercise in the resistance trials. These differences could be due to the lower energy expenditure experience during resistance exercise or to less gut disturbances in resistance *vs.* aerobic exercise. A 2014 meta-analysis by Schubert and colleagues [37] found that studies involving exercise with greater energetic and mechanical demands (*i.e.*, running) tended to report greater appetite suppression. The authors speculated that this might be due to altered splanchnic blood flow inhibiting ghrelin secretion [37]. Thus, an acute bout of aerobic exercise may have a greater impact on circulating hormones; especially gut hormones, compared to an acute bout of resistance exercise.

**Table 2:** Studies examining the effect of acute aerobic exercise on subjective and objective appetite and energy intake in exercise-trained individuals.

Reference	Population ^a^	Intervention	Outcomes		
			Appetite Hormones	Subjective Appetite	Energy Intake (Relative or Absolute)
*Studies in Exercise-Trained Women*					
Larson-Meyer *et al.* 2012 [22]	Female runners (*n* = 9) Age = 23.7 ± 2.4 BMI = 19.8 ± 1.0 VO_2_max = 49.7 ± 3.0	60-min 70% VO_2_max run or walk	Acylated ghrelin (*p* = 0.075), PYY (*p* < 0.01) and GLP-1 (*p* = 0.022) were ↑ post-exercise *vs.* rest.	No difference in hunger ratings between exercise/rest conditions.	No difference in absolute EI between conditions. Relative EI was ↓ post-exercise *vs.* rest (*p* = 0.001).
Pomerleau *et al.* 2004 [48]	Females (*n* = 13) Age = 22.2 ± 2.0 BMI = 22.2 ± 2.4 VO_2_max = 44.0 ± 4.7	Walking on treadmill until 350 kcal expended: VO_2_max 40% = Low, 70% = High Intensity (HI).	NR	No difference in appetite between conditions.	Absolute EI ↑ after HI in post-exercise meal (*p* < 0.02). Relative EI ↓ post-exercise in HI/Low conditions *vs.* (*p* < 0.001)
*Studies in Exercise-Trained Men*					
Balaguera-Cortes *et al.* 2011 [14]	Males (*n* = 10) Age = 21.3 ± 1.4 BMI = 23.7 ± 2.0 VO_2_peak = 58.1 ± 7.3	Treadmill running for 45 min at 70% VO_2_peak.	Acylated ghrelin ↓ post-exercise *vs.* rest (*p* = 0.05).	NR	No influence on absolute EI.
Becker *et al.* 2012 [31]	Males (*n* = 8) Age = 28 ± 2 BMI = 24 + 0.9 VO_2_max = 54.9+2.6	Cycling for 60 min at 70% VO_2_max.	Acylated ghrelin ↓ post-exercise *vs.* control (*p* = 0.04).	Appetite ↓ post-exercise *vs.* control (*p* = 0.07).	NR
Broom *et al.* 2007 [13]	Males (*n* = 9) Age = 21.1 ± 0.3 BMI = 23.1 ± 0.4 VO_2_max = 62.1 ± 1.8	Running 60 min at 72% VO_2_max; rest for 8 h post-exercise; test meal 3 h post-exercise.	Acylated ghrelin was ↓ h post-exercise *vs.* rest (*p* < 0.05)	Appetite was ↓ 3 h post-exercise *vs.* rest (*p* < 0.05)	NR
Broom *et al.* 2009 [15]	Males (*n* = 11) Age = 21.1 ± 0.3 BMI = 23.1 ± 0.4 VO_2_max = 62.1 ± 1.8	Treadmill running 60 min at 70% VO_2_max	Acylated ghrelin ↓ post-exercise (*p* < 0.05) PYY ↑ post-exercise (*p* < 0.05)	Appetite was ↓ post-exercise *vs.* rest (*p* < 0.05)	NR
Deighton *et al.* 2012 [44]	Males (*n* = 12) Age = 23 ± 3 BMI = 22.9 ± 2.1 VO_2_max = 57.5 ± 9.7	Treadmill running 60 min at 70% VO_2_max; fasted *vs.* 4–5 h post-prandial	NR	Appetite was ↓ in both trials (*p* < 0.05); greater ↓ post-prandial *vs.* fasted (*p* < 0.05)	No difference in EI.
Dieghton, Karra, Batterham, Stensel 2013 [32]	Males (*n* = 12) Age = 22 ± 3 BMI = 23.7 ± 3 VO_2_max = 52.4 ± 7.1	Cycling: steady-state (SS) 60 min at 59.5% ± 1.6% VO_2_max. High-intensity (HI): 10 times for 4 min intervals at 85.8% ± 4% VO_2_max with 2 min rests.	PYY_3–36_ ↑ post-exercise in SS & HI (*p* = 0.002, *p* = 0.015, respectively)	Appetite ↓ post-exercise, with ↑ suppression in HI (*p* < 0.05).	Relative EI ↓ post-exercise *vs.* control (*p* < 0.005).
Imbeault *et al.* 1997 [72]	Males (*n* = 11) Age = 24.2 ± 3.3 BMI = 23.2 ± 2.3 VO_2_max = 56.7 ± 5	Low-intensity (Low): walking at 35% VO_2_max for 490 kcal (72 ± 14 min). High-intensity (HI): run at 75% VO_2_max for 490 kcal (34 ± 6 min).	NR	Appetite not significantly different between treatments.	Relative EI ↓ after HI *vs.* control (*p* < 0.001) and Low intensity (*p* < 0.05)
Kawano *et al.* 2013 [18]	Males (n = 15) Age = 24.4 ± 1.7 BMI = 22.1 ± 2.0 VO_2_max = 47.0 ± 6.2	Rope skipping 3 times for 10 min with 5 min rest at 64.8% ± 6.9% VO_2_max; Cycling 3 times for 10 min with 5 min rest at 63.9% ± 7.5% VO_2_max.	Acylated ghrelin ↓ up to 30 min post-exercise (*p* < 0.0167); PYY_3–36_ ↑ immediately post-exercise (*p* < 0.0167)	Appetite ↓ post-exercise (*p* < 0.0167). Appetite ↓ during rope skipping *vs.* cycling (*p* < 0.0167)	NR
Kelly *et al.* 2012 [19]	Males (*n* = 10) Age = 21.4 ± 1.3 BMI = 23.94 ± 2.1 VO_2_peak = 59.8 ± 8.6	Treadmill running for 45 min at 70% VO_2_peak in hydrated (HY) or dehydrated (DH).	Post-exercise, acylated ghrelin ↓ in DH *vs.* control (*p* = 0.045) and HY (*p* = 0.014).	No difference in appetite between trials.	Relative EI ↓ post-exercise (*p* < 0.001).
King, Wasse, Broom, Stensel 2010 [73]	Males (*n* = 14) Age = 21.9 ± 0.5 BMI = 23.4 ± 0.6 VO_2_max = 55.9 ± 1.8	Brisk walking 60 min at 45.2% ± 2% VO_2_max.	No difference in acylated ghrelin between trials.	No difference in appetite between trials.	Relative EI ↓ post-exercise (*p* < 0.001).
King *et al.* 2010 [20]	Males (*n* = 9). Age = 22.2 ± 0.8 BMI = 23.6 ± 0.4 VO_2_max = 60.5 ± 1.5	Treadmill running for 90 min at 68.8% VO_2_max.	Acylated ghrelin ↓ exercise trial (*p* < 0.0045); trend ↓ post-exercise (NS).	Appetite ↓ with exercise *vs.* control (*p* < 0.05).	No compensatory ↑ in EI, despite ↑ EE (*p* < 0.001).
King *et al.* 2011 [21]	Males (*n* = 12) Age = 23.4 ± 1.0 BMI = 22.8 ± 0.4 VO_2_max = 57.3 ± 1.2	Treadmill running at 70% VO_2_max for 90 min in exercise energy deficit (ED), food deficit (FD) or control.	Acylated ghrelin ↓ post-exercise (*p* < 0.05), ED ↑ PYY_3–36_ post-exercise (*p* < 0.05).	Appetite was ↑ after FD *vs.* ED (*p* < 0.05). No difference between ED and control.	No compensatory ↑ in EI, despite ↑ EE in the ED. EI ↑ in FD *vs.* ED (*p* < 0.05).
Shorten *et al.* 2009 [45]	Males (*n* = 11) Age = 20.8 ± 2.1 BMI = 24.1 ± 2.3 VO_2_peak = 53.8 ± 8.9	Treadmill running at 70% VO_2_peak for 40 min at neutral (25 °C) or in heat (36 °C).	PYY ↑ post-exercise (*p* < 0.05) in heat and neutral conditions.	NR	Relative EI ↓ post-exercise in heat (*p* = 0.002), similar between neutral and control (rest at 25 °C).
Ueda *et al.* 2009 [27]	Males (*n* = 10) Age = 23.4 ± 4.3 BMI = 22.5 ± 1.0 VO_2_max = 45.9 ± 8.5	Cycling 30 min at 75% or 50% VO_2_max or rest.	Exercise ↑ (*p* < 0.01) PYY_3–36_ & GLP-1. PYY_3–36_ ↑ in 75% *vs.* 50% VO_2_max at 60 min post-exercise (*p* < 0.01).	Appetite was ↓ post-exercise in exercise *vs.* rest (*p* = 0.045).	EI ↓ post-exercise *vs.* rest (*p* < 0.01).
Vatansever-Ozen *et al.* 2011 [28]	Elite male soccer players (*n* = 10) Age = 20.12 ± 0.17 BMI = 22.03 ± 0.44 VO_2_max = 62.74 ± 5	Treadmill running 105 min at 50% VO_2_max, then 15 min 70% VO_2_max.	Acylated ghrelin ↓ 120, 180, 240 min post-exercise (*p* < 0.05)	Appetite ↓ 120, 180, 240 min post-exercise (*p* < 0.05)	Relative EI ↓ in post-exercise *vs.* rest (*p* = 0.018)
Wasse *et al.* 2012 [29]	Males (*n* = 10) Age = 24 ± 3 BMI = 24.8 ± 2.4 VO_2_max = 56.9 ± 6.5	Treadmill running 60 min 70% VO_2_max at normoxic (20.9% O_2_) or hypoxic (12.7% O_2_).	Acylated ghrelin ↓ post-exercise (*p* = 0.01); PYY ↑ (*p* = 0.04) in both conditions.	Appetite ↓ post-exercise (*p* < 0.001).	Relative EI ↓ post-exercise (*p* < 0.001).
Wasse *et al.* 2013 [34]	Males (*n* = 12) Age = 22.7 ± 2.3 BMI = 23.4 ± 2.4 VO_2_max (running) = 57.8 ± 9.9 VO_2_max (cycling) = 50.0 ± 9.5	Exercise trials: running and cycling for 60 min at 70% VO_2_max.	Acylated ghrelin ↓ post-exercise (*p* < 0.05).	No differences in appetite between trials.	NR
Zoladz *et al.* 2005 [74]	Males (*n* = 8) Age = 23 ± 0.5 BMI = 22.42 ± 0.49 VO_2_max = 51.6 ± 1.5	Incremental cycling in fed or fasted state until exhaustion or 150 Watts (59 ± 2%VO_2_max)	No change in total ghrelin; Gastrin ↓ at 150 Watts in fed condition (*p* = 0.008).	NR	NR
*Combined Studies with Exercise-Trained Men & Women*					
Burns *et al.* 2007 [75]	Males (*n* = 9) Age = 24.5 ± 1.3 BMI = 23.4 ± 1 VO_2_max = 63.2 ± 2.5 Females (*n* = 9) Age = 25.1 ± 1.2 BMI = 22.5 ± 0.8 VO_2_max = 52.1 ± 2.4	Treadmill running for 60 min at 73.5% VO_2_max	No difference in total ghrelin post-exercise compared to control trail.	Post-exercise appetite ↓ for 60 min (*p* = 0.009)	NR
O’Connor *et al.* 1995 [26]	Marathon runners: Males = 23; Female = 3 Age = 37 years (range = 19–61 years)	Marathon running: Average time = 239 min	GLP-1 & PPY ↑ post- & 30 min post-race (*p* < 0.01).	NR	NR
Laan *et al.* 2010 [71]	Males and females (*n* = 19) Age = 22.3 ± 2.5 BMI = 22.5 ± 1.8 VO_2_max = 60.1 ± 22.5	Cycling 35 min at 70% HRR.	NR	Post-exercise appetite ↓ (*p* = 0.03).	Relative EI ↓ post-exercise *vs.* rest (*p* = 0.003).
Russell *et al.* 2009 [47]	Endurance runners: Males (*n* = 11) Age = 27 ± 9, BMI = 21.9 ± 1.5 VO_2_max = 63.7 ± 6.3 Females (*n* = 10) Age = 29 ± 7 BMI = 21.0 ± 1.1 VO_2_max = 53.2 ± 5.4	8-day session: 7-day running 90 min at 63% VO_2_max + 1-day 10 kilometer time trial	Total ghrelin and PYY ↑ immediately post-exercise (*p* < 0.0001).	NR	NR

^a^ Age in years, BMI (kg/m^2^) = Body Mass Index, VO_2_max (mL/kg/min) = maximal oxygen uptake, PYY = Peptide YY, GLP-1 = Glucagon-like Peptide 1, VAS = Visual Analog Scale, EI = energy intake, TDEE = total daily energy expenditure, MOD = moderate, VIG = vigorous, RMR = resting metabolic rate, HRR = heart rate reserve, RE = resistance exercise, 1RM = one repetition max, HRmax = heart rate max, PA = physical activity, EB = energy balance, HI = high intensity exercise, NR = not reported.

### 4.2. Exercise Intensity

The intensity of exercise can also impact subjective and objective measures of appetite. Studies examining moderate-intensity exercise (MIE) (~40%–60% VO_2_max) *vs.* high-intensity exercise (HIE) (~>60% VO_2_max) show an intensity-dependent influence of exercise on appetite [27,32,48]. Ueda and colleagues [27] showed that exercise-trained males (VO_2_max = 45.9 mL/kg/min) cycling at a HIE (75% VO_2_max) had a greater rise in PYY compared to cycling at MIE (50% VO_2_max). However, they found no differences in GLP-1 between exercise trials [27]. Similarly, Deighton and colleagues [32] found increased PYY following HIE intermittent cycling (85.8% VO_2_max) compared to MIE cycling (59.5% VO_2_max) in healthy, exercise-trained males (VO_2_max = 52.4 mL/kg/min). The high- and moderate-intensity exercises were matched for energy expenditure, and thus appetite suppression was a result of the exercise intensity [32]. Conversely, when Pomerleau *et al.* [48], examined the impact of low-intensity (40% VO_2_peak) and HIE (70% VO_2_peak) walking in exercise-trained women (VO_2_max = 44 mL/kg/min; 30–45 min, three to five times/week), they saw no difference in subjective appetite measures. As expected, HIE increased absolute energy intake (*p* < 0.02), while relative energy intake was higher in both the HIE and low-conditions *vs.* rest (*p* < 0.001) [48]. They did not measure appetite-regulating hormones.

Inconsistent results between studies can be attributed to differences in methods, gender, and level of subject training status. Two recent meta-analyses by Schubert and colleagues [37,39] reported that despite a wide range of exercise intensities (35%–80% VO_2_max), few studies have examined the effect of exercise on appetite-regulating hormones at intensities >75% VO_2_max. It has yet to be determined the exercise intensity threshold that leads to greater appetite suppression. Therefore, more research is needed to fully understand the influence of exercise intensity on subjective and objective measures of appetite.

### 4.3. Sex Differences

The impact of exercise on appetite has also been compared between men and women [17,37]. Hagobian *et al.* [16] compared previously sedentary, overweight/obese men and women running at 50%–65% VO_2_peak for 83–89 min (30% of total daily energy expenditure). They found that exercise increased acylated ghrelin (stimulating appetite) in the women, but not in the men [16]. Thus, overweight/sedentary women may be more likely to experience post-exercise appetite stimulation compared to male counterparts. However, in 2013 Hagobian *et al.* [17] compared habitually active men and women (>3 h/week aerobic exercise, men: VO_2_peak = 42.9 ± 6.5 mL/kg/min, women: VO_2_peak = 39.9 ± 5.5 mL/kg/min) and found no difference in appetite or appetite-regulating hormones between sexes cycling at 70% VO_2_peak for 82–86 min. In this study, both sexes experienced post-exercise appetite suppression in response to acute endurance exercise. Thus, it is not clear whether sex differences exist, and if differences depend on fitness level and body composition. To date, no study has compared the appetite response to high-intensity exercise in highly fit men and women.

### 4.4. Exercise Training

What impact does exercise training have on appetite? Currently, only four studies have investigated the influence of exercise training on measures of appetite and appetite-regulating hormones [16,24,25,76] (see Table 3). These studies examined the initiation of exercise in previously sedentary and/or overweight/obese subjects. Thus, the effects of exercise training on appetite may be different than that of active, exercise-trained individuals who regularly engage in moderate or high-intensity exercise. Based on this limited research it appears that improving one’s general fitness level does influence appetite and hormonal response, but the direction and magnitude are yet to be determined.

**Table 3:** Review of studies examining the effect of exercise training on subjective and objective appetite in sedentary and/or overweight individuals participating in an exercise-training program.

Reference	Population ^a^	Intervention	Outcomes	
			Appetite Hormones	Subjective Appetite
*Exercise Training*				
Guelfi *et al.* 2013 [76]	Overweight/obese males (*n* = 33) Age = 49 ± 7 BMI = 30.8 ± 4.2	12-week training (3 day/week). 3 groups: Aerobic (*n* = 12) 40–60 min at 70%–80% HRmax, Resistance (*n* = 13) 3–4 sets, 8–10 reps at 75%–85% 1RM, Control (*n* = 8)	No change in acylated ghrelin or PYY after 12-week aerobic or resistance training program.	Perceived fullness was higher after the aerobic training program. No diff in resistance or control.
Hagobian *et al.* 2009 [16]	Overweight: Males (*n* = 9) Age = 26.81 ± 1.8 BMI = 25.7 ± 2.3; VO_2peak_ = 44.9 ± 4.8. Females (*n* = 9) Age = 23.3 ± 8 BMI = 28.0 ± 3.5 VO_2peak_ = 34.9 ± 5.2.	Treadmill running 50%–65% VO_2peak_ until 30% of TDEE in DEF or BAL conditions (crossover).	Females: ↑ acylated ghrelin after training (*p* < 0.05). Males & females: insulin ↓ after training (*p* < 0.05).	Males: Appetite was ↓ in BAL *vs.* DEF condition (*p* < 0.05). Females: No difference in appetite between conditions.
Martins, Kulseng, King, Holst, Blundell 2010 [25]	Sedentary overweight males (*n* = 8) & females (*n* = 7) Age = 36.9 ± 8.3 BMI = 31.3 ± 2.3 VO_2_max = 32.9 ± 6.6.	12-week training (5 day/week): Treadmill walking or running at 75% HRmax until 500 kcal energy deficit.	Ghrelin ↑ after 12-week training (*p* < 0.05).	Appetite ↑ after 12-week training (*p* < 0.0001).
Martins *et al.* 2007 [24]	Sedentary subjects: Males (*n* = 11) Age = 29.8 ± 11.6 BMI = 23.4 ± 2.4 VO_2_max = 32.7 ± 5.1 Women (*n* = 14) Age = 29.8 ± 11.6 BMI = 22.1 ± 2.2, VO_2_max = 29.9 ± 4.3	6-week training: cycling 30–45 min 4 times per week, 65%–75% HRmax. High-energy (HEP)/low-energy preloads (LEP) were before test buffet pre/post 6-week training.	NR	Appetite ↓ after HEP in men after 6-week training (*p* < 0.01) but not in women.

^a^ Age in years, BMI = Body Mass Index (kg/m^2^), VO_2_max in mL/kg/min, TDEE = total daily energy expenditure, DEF = deficit, BAL = balance, NR = not reported.

## 5. Exercise and Energy Intake

There is strong evidence supporting changes in appetite and appetite-regulating hormones following exercise. However, the impact of these changes may or may not result in subsequent changes in energy intake, either the relative or absolute number of calories consumed. Laboratory based studies assessing energy intake are difficult to interpret because they are typically short-term (e.g., two-24 h post-exercise) and do not simulate real life. Most studies that measure energy intake do so using an *ad libitum* food buffet approach [19,20,21,22,28,29,32,45,48,71,72,73] (see Table 2). Although this method has some limitations, such as types of food offered, eating environment, and possibility promoting over consumption, it has been shown to be reproducible for measuring energy intake under sedentary and post-aerobic and resistance exercise conditions [19,20,21,22,28,29,30,45,48,71,72,73]. However, the question remains as to how well this method replicates what happens outside the laboratory. Conversely, in free-living conditions other factors, such as food selection or social influences, may impact energy intake.

Despite few studies reporting changes in absolute energy intake following exercise, relative energy intake appears to be influenced by exercise in exercise-trained individuals. Absolute energy intake refers to the total the amount of energy (kcals) consumed post-exercise, whereas relative energy intake takes into account the amount of energy expended in the exercise bout. Relative energy intake is calculated as energy intake in the post-exercise period minus the total amount of energy expended in the exercise bout. In a meta-analysis by Schubert and colleagues [39], the authors concluded that 30–120 min of aerobic exercise did create a short-term energy deficit (exercise energy expenditure ranging from 406–772 kcals). They found that despite most studies showing no effect of exercise on absolute energy intake (*n =* 51 trials; 41 kcal increase), exercise does have a large effect on relative energy intake (*n =* 25 trials; >119 kcal decrease). Thus, energy intake was not fully compensated for by the increased energy expenditure created by the exercise [39]. As shown in Table 2, there were 13 studies with a transient suppression of either relative or absolute energy intake post-exercise in exercise-trained males and females [19,20,21,22,27,28,29,32,45,48,71,72,73]. Thus, post-exercise energy intake may not be fully compensated for in these individuals, contributing to their maintenance of normal body weight. In a recent study by Jokisch *et al.* [77] comparing exercise-trained to non-trained individuals, researchers found that healthy normal weight active males (438 min/week physical activity; 12.5% body fat) cycling for 45 min at 65%–75% HRmax were better able to match energy intake to energy expenditure compared to their non-trained counterparts (32 min/week of physical activity; 15% body fat). Thus, highly fit individuals who regularly engage in exercise do not overcompensate for energy expended during exercise. In contrast, individuals who engage in lower intensity exercise (low to moderate) are more responsive to the appetite-stimulatory effects of exercise [39]. This evidence suggests that regular acute exercise may keep athletes from overeating, preventing weight gain in those accustomed to exercise [28].

## 6. Diet and Appetite in Active Women

Exercise can decrease subjective feelings of hunger and increase overall hormonal suppression of appetite (See Table 2). In exercise-trained men, most studies (*n =* 12) show a suppressive effect of exercise on appetite-regulating hormones, subjective appetite ratings (*n =* 8), and subsequent absolute or relative energy intake (*n =* 8). For exercise-trained women the data are limited and equivocal. Only two studies have investigated the effect of exercise on appetite response in exercise-trained females [22,48] (See Table 2). Of these studies, only one measured appetite-regulating hormones [22]. In this study, Larson-Meyer and colleagues [22] measured acylated ghrelin, PYY, and GLP-1 in female runners (*n =* 9) and walkers (*n =* 10) and found all hormones to be elevated post-exercise (60 min at 70% VO_2_max). Relative energy intake in the post-exercise meal was negative (−194 ± 206 kcal) following running compared to walking (41 ± 196 kcal), while both were suppressed compared to rest [22]. These results, with the exception of elevated concentrations of acylated ghrelin after exercise, are similar to what is observed in exercise-trained males. An additional consideration in exercise-trained women is the influence of energy restraint on energy intake. In a recent study on the influence of exercise on energy intake in exercise-trained women, Pomerleau and colleagues [48] found that five out of 13 subjects showed energy restraint; however, there were no differences in energy intake between the two groups.

It is yet to be determined if males and females exhibit the same hormonal response following exercise. As indicated earlier, Hagobian and colleagues [16,17] found conflicting results between men and women. When they compared overweight/obese men and women, the women had higher levels of hunger and hormonal responses compared to the men [16]. However, they saw no differences in sexes when they compared subjective and objective appetite and energy intake in healthy, normal weight males and females after exercises [17]. The inconsistency in these results may be due to differences in subject characteristics, since overweight/obese individuals may exhibit a different response to exercise than healthy or exercise-trained individuals. Nonetheless, the influence of exercise on appetite in women is still largely unknown. In addition, no research has compared the impact of appetite or gut hormones in exercise-trained men and women.

## 7. Diet

Female athletes and exercise-trained women are often preoccupied with body weight and shape, both for performance and aesthetic reasons [78]. As a result, they may engage in dietary behaviors to control weight, such as energy restriction or the elimination of food groups. Another option may be the consumption of low-ED foods, which are high in fiber and low in calories.

### 7.1. Low-Energy Dense Diets

The energy density of food is defined as the energy (kcal) per gram (g) of food. See Table 4 for examples of foods that are classified as low-energy density, include whole fruits and vegetables, beans and legumes, whole grains, and broth/vegetable-based soups. A low-ED food, such as an apple, has an energy density of 0.61 kcal/g and is higher in volume and lower in kcals/g than a high-ED food, such as potato chips (energy density = 5.43 kcal/g). Thus, low-ED diets are high in whole fruits and vegetables, whole grains, especially wet grains such as brown rice and oatmeal, and low-fat protein foods (e.g., beans and reduced fat dairy/meats). These diets also eliminate or dramatically reduce high calorie sweetened beverages.

Overall, a low-ED diet can have similar weight of food (g/day) as a high-ED diet but the diet will be lower in total energy (kcal/day). Ello-Martin *et al.* [79] showed that low-ED diets will increase satiety leading to reduced energy intake, which ultimately contributes to weight loss. Individuals also rely more on external cues indicating the volume of food consumed, rather than the energy content of the food, for satiation. For example, Bell and colleagues [80] allowed 18 healthy, normal-weight, sedentary female participants to consume food *ad libitum* from three diets with high-, medium- or low-energy density. Those consuming the high-ED diet consumed significantly more energy per day (1800 ± 86 kcal/day) than those in the medium-ED (1519 ± 67 kcal/day) or low-ED (1376 ± 43 kcal/day) diets. None of the diet groups reported a difference in appetite, thus, indicating that visual cues of volume effect intake more than energy content [80].

**Table 4:** Energy density (kcal/g) classification of commonly consumed foods.

Low-Energy Dense Foods	High-Energy Dense Foods
Apple, raw, with skin (0.61 kcal/g)	Potato chips (5.43 kcal/g)
Carrots, baby, raw (0.39 kcal/g)	Peanut butter, smooth (5.87 kcal/g)
Lettuce, green leaf, raw (0.16 kcal/g)	Swiss cheese, slice (3.79 kcal/g)
Oatmeal, regular, cooked (0.85 kcal/g)	Raisins, seedless (3.00 kcal/g)
1% Cottage cheese (0.87 kcal/g)	Sirloin steak (2.12 kcal/g)

United States Department of Agriculture (USDA) National Nutrient Database for Standard Reference [81].

For the majority of the population, consuming a low-ED diet is recommended because it is high in whole, unprocessed foods that are lower in calories, fat, refined sugar and salt. This type of a diet promotes a healthy weight, reduces chronic disease risk, and is sufficient to fuel energy needs [82]. However, for athletes and highly exercise-trained individuals, consuming a low-ED diet may not provide sufficient energy to fuel health, activities of daily living, and exercise workouts.

### 7.2. Low-Energy Diets and Exercise

Low-ED diets can suppress appetite and reduce overall energy intake. However, research in this area has been limited to active endurance trained women, who typically need to be lean to perform well in their sport. For exercise-trained women with high-energy needs, consuming a low-ED diet may increase satiety or fullness before energy needs are met. The overall result can be a lower overall energy intake at a time when exercise energy demands are high. Chronic negative energy balance in women who participate in high levels of exercise increases the risk for developing exercise-related menstrual dysfunction (ExMD) [41] and the associated negative health consequence, including increased risk of poor bone health, injury and stress fractures, illness, impaired nutritional status, and decreased exercise performance [41].

Only two studies have examined the relationship between consuming a low-ED diet and markers of long-term health, such as menstrual status and risk for injury [83,84]. Reed and colleagues [83] found that women with ExMD consumed diets that were significantly lower in energy density (0.77 ± 0.06 kcal/g) than their eumenorrheic counterparts (1.06 ± 0.09 kcal/g). They included all beverages in their calculation of energy density [83]. Energy density can be calculated with or without the inclusion of beverages [85]. Typically, beverages such as water, diet and regular soda, alcohol, juice and milk, are not included in the calculation because they do not have the same impact on appetite as whole food. Including these beverages may show a disproportionately low-energy density that may not be reflective of the actual diet. Finally, exercise-trained women may have high beverage intakes to replace fluids lost in exercise. Hand *et al.* [84] also compared the dietary energy density using seven-day food records from female athletes categorized with ExMD (*n* = 8, age = 22.6 ± 3.3 years) and eumenorrheic controls (*n* = 9, age = 23.1 ± 4.3 years). Energy density calculations included only food and liquid meal-replacement beverages (Gatorade Nutrition Shake), and excluded caloric beverages such as alcohol, juice and milk. Energy density was lower in the ExMD (mean = 1.6 ± 0.2 kcal/g)* vs.* the eumenorrheic athletes (mean = 1.8 ± 0.3 kcal/g) (*p* = 0.089), but differences were not significant. All participants had low intakes of alcohol and juice. For both groups, the overall energy density of the diet was classified as medium (1.5–4.0 kcal/g), with the ExMD group at the low-end of this range. For the ExMD group, 50% of the women consumed a low-ED diet (<1.5 kcal/g), while none of the eumenorrheic group consumed a low-ED diet. Further research needs to examine the energy density of diets consumed by exercise-trained women, using appropriate methods to calculate energy density, to determine if low-ED diets in exercise-trained women with ExMD are contributing to their low-energy intakes and ExMD.

As discussed earlier, exercise may transiently suppress appetite. Although this suppression of appetite may be short [23], the post-exercise period is an important time for replenishing glycogen stores and providing protein for muscle building and repair [86]. If appetite is reduced during the first 30–45 min post-exercise, there is an increased probability that an athlete will not consume adequate energy. Thus, convenient, palatable, high-ED foods providing adequate amounts of carbohydrate and protein are important to consume post-exercise.

### 7.3. Other Potential Mechanisms for Appetite Suppression in Active Individuals

Highly fit individuals who participate in high-intensity exercise often do not feel hungry post-exercise. Besides the impact of exercise on appetite-regulating hormones, other factors may work independently or synergistically to delay hunger post-exercise in these individuals. Other potential factors that may perturb normal metabolism and acutely suppress appetite in exercise-trained individuals include extreme environmental conditions (altitude, heat), elevated core body temperature, fatigue, dehydration, and gastrointestinal stress [34]. Crabtree *et al.* [87] found that strenuous exercise suppressed appetite while increasing preference for low-calorie over high-calorie foods. In addition, exercise increases core body temperature, thirst, and circulating appetite-regulating hormones [87]. Thus, after a hard effort an athlete may not feel hungry and when they do eat select lower calorie foods. They many even choose rest over eating. Suppressed post-exercise appetite due to changes in gut hormones along with factors such as elevated core body temperature, dehydration, and gastrointestinal distress, may work synergistically and delay hunger for more than 24 h.

## 8. Conclusions

Exercise is an important part of a healthy lifestyle. In addition to directly increasing energy expenditure, exercise may also influence appetite and energy intake. Recent studies have shown that an acute bout of exercise has the capacity to alter circulating appetite-regulating hormone concentrations [37]. Further, exercise intensity may also influence the degree of appetite suppression. HIE has been shown to lead to greater appetite suppression than low- or moderate-intensity exercise [27]. However, most research, examining the effect of exercise on appetite and appetite-regulating hormones, has been done in exercise-trained males. Thus, the impact of exercise on appetite in exercise-trained, normal- or lean-weight women is limited. Female athletes who participate in sport are often concerned about body aesthetics and weight, thus, understanding the influence of exercise and diet on appetite and overall energy balance is important [88]. Diet can also influence appetite and subsequent energy intake. When HEI exercise in combined with a low-ED diet, total energy intake may be further decreased. Although, consuming a low-ED diet has many health benefits, for some highly exercise-trained individuals, this eating pattern may not provide enough energy and nutrients during periods of heavy exercise training. In this population, a low-ED diet can lead to chronically low-energy intake and increased risk for negative health consequences. More research is needed, especially in active women, examining the combined effect of diet and exercise on appetite regulation in active individuals.
